# Retropharyngeal hematoma in the context of obstructive sleep apnea: a case report and review of the literature

**DOI:** 10.1186/s13256-019-2202-9

**Published:** 2019-08-24

**Authors:** Christian Warken, Nicole Rotter, Joachim Theodor Maurer, Ulrike Attenberger, Anne Lammert

**Affiliations:** 1 Department of Otolaryngology, Head and Neck Surgery, University Medical Center Mannheim, Medical Faculty Mannheim, Heidelberg University, Mannheim, Germany; 2 Institute of Clinical Radiology and Nuclear Medicine, University Medical Center Mannheim, Medical Faculty Mannheim, Heidelberg University, Mannheim, Germany

**Keywords:** Retropharyngeal hematoma, Obstructive sleep apnea, OSA, Dyspnea, Hemorrhage

## Abstract

**Background:**

Obstructive sleep apnea is related to increased systemic inflammation and arterial hypertension. We present a case of retropharyngeal hematoma without trauma und hypothesize that this could be caused by untreated obstructive sleep apnea.

**Case presentation:**

A 47-year-old white woman with unilateral pharyngeal discomfort presented to our ear, nose, and throat clinic. She had no risk factors for the development of a spontaneous retropharyngeal hematoma, for example, hypertension or coagulation disorder. As she was overweight, the anamnesis included signs of obstructive sleep apnea such as snoring or breathing arrests during the night, which she denied. An endoscopic examination showed a submucosal hemorrhage of the posterior wall of her pharynx. Magnetic resonance imaging revealed a retropharyngeal hematoma without evidence of the injury of any blood vessel. A subsequent seven-channel polygraphy revealed a severe obstructive sleep apnea with an apnea-hypopnea index of 59.5 per hour. She was subsequently treated with auto-titrating continuous positive airway pressure resolving obstructive sleep apnea immediately. Two months after this episode she presented without any complaints.

**Conclusion:**

In consequence of this case we are convinced that an untreated obstructive sleep apnea can lead to retropharyngeal hematoma.

## Background

A sore throat, dyspnea, and dysphagia are common symptoms among patients presenting in an ear, nose, and throat (ENT) clinic. Most common causes are local infections such as pharyngitis [1]. Less common causes are neoplasms or abscesses of the retropharyngeal space. Rare causes are an ectopic running internal carotid artery, a degenerated spine, or several systemic diseases like sarcoidosis [2]. Dyspnea can also be caused by pulmonary or cardiac diseases [3].

Obstructive sleep apnea (OSA) is a common disease. In Western industrial countries the prevalence of OSA is approximately 6% among women and 13% among men, showing an increase of 14–55% during the last 20 years [4]. The leading symptoms of OSA are loud irregular snoring and non-restorative sleep resulting in excessive daytime sleepiness, and reduced daytime performance and quality of life. Accessory symptoms are nycturia, headache, intellectual deterioration, memory disorders, depression, mouth dryness, and decreased libido. Many patients with OSA are undiagnosed or untreated. Consequences of untreated OSA can be, for example, hypertension, heart disease, stroke, and an abnormal glucose tolerance [5].

To the best of our knowledge this is the first case report describing a retropharyngeal hematoma most likely caused by untreated OSA.

## Case presentation

**Fig. 1:**
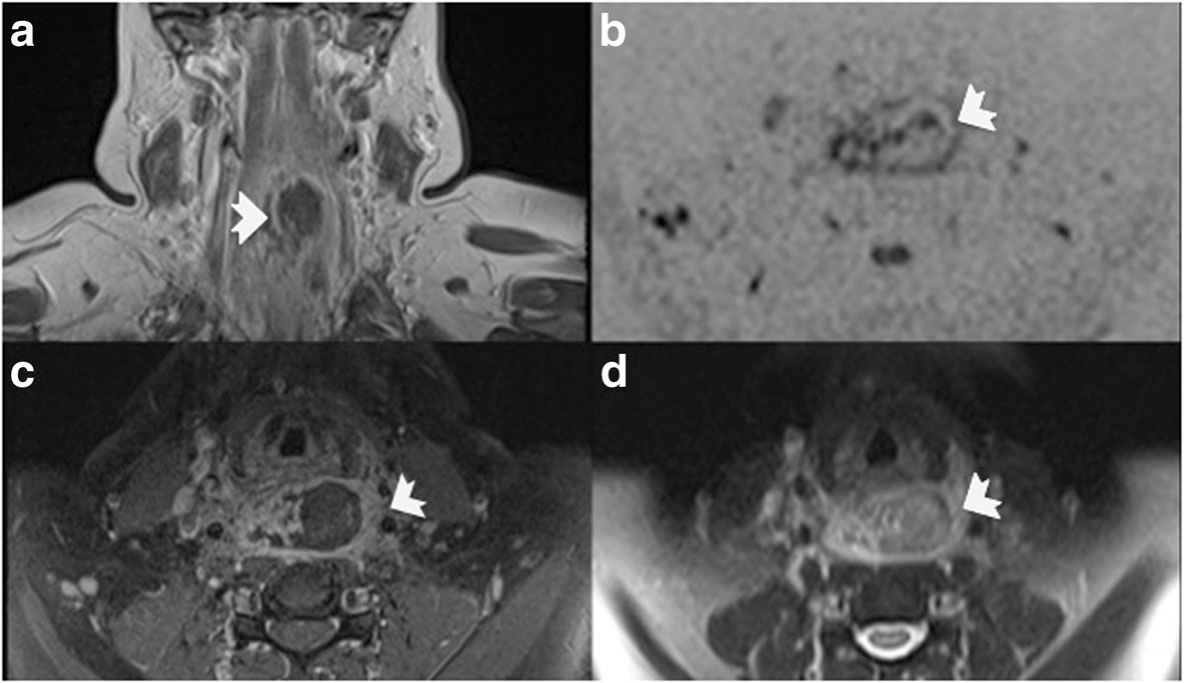
Magnetic resonance imaging of retropharyngeal hematoma. **a** A lesion with rim enhancement (*arrow*) is displayed on a T1-weighted coronal image. **b** Diffusion restriction can be found at the margins of the lesion (*arrow*). **c** and **d** T1-weighted fat suppressed and T2-weighted images accordingly show a lesion with a central core, surrounded by an enhancing rim. The intermediate hyperintense signal on T2-weighted (*arrow*) image indicates hematoma

A 47-year-old white woman was admitted to our ENT clinic with the suspicion of acute gangrenous pharyngitis. She presented with unilateral pharyngeal discomfort starting 2 days before and precipitating during the previous 3 hours. Later, she also reported dysphagia, position-dependent dyspnea, and a superficial neck swelling. She had no history of trauma, no known coagulopathy, and had not been taking any anticoagulants. Arterial hypertension and obesity were part of her pre-existing conditions. She denied any snoring or any observed apneas. However, her mother was diagnosed as having OSA and was treated by continuous positive airway pressure (CPAP). An endoscopic examination showed a submucosal hemorrhage of the posterior wall of her pharynx, from the nasopharynx to the hypopharynx and extending to her vocal cords. The parameters of her hemostasis were standard with an activated partial thromboplastin time of 21.4 seconds and thrombocytes of 216 per nanoliter. A magnetic resonance imaging (MRI) revealed a retropharyngeal/retrolaryngeal hematoma, measuring 4 × 2.7 cm without any evidence of injury of any blood vessel. An orotracheal intubation was indicated but rejected by our patient. She was referred to the intensive care unit (ICU) for further monitoring. After 1 day she was moved to a general ward. As snoring was reported by the nurses of the ICU, a subsequent seven-channel polygraphy was conducted, showing an apnea-hypopnea index of 59.5 per hour with an oxygen desaturation index of 69.9 per hour. She was subsequently provided with an automatic CPAP device to treat the newly diagnosed OSA. Under further monitoring the hematoma regressed spontaneously. After 6 days she could be discharged (Fig. 1).

Two months after this episode, she presented without any complaints. The OSA remained sufficiently treated every night since the initial stay. A physical examination showed normal findings. There was no evidence of a recurrent retropharyngeal hematoma.

## Discussion and conclusions

Today we know that there are many causes of retropharyngeal hematoma, including retropharyngeal abscess, trauma, sudden pressure changes (due to vomiting, coughing and sneezing), foreign body ingestion, surgery, central venous cannulation and carotid aneurysm, portal hypertension, the presence of tumors, and an aberrant artery at the thoracic inlet. Also, spontaneous bleedings in association with anticoagulant therapy or bleeding diatheses are possible causes [6–8].

The retropharyngeal space is an area of loose connective tissue. The buccopharyngeal fascia which surrounds the pharynx, trachea, esophagus, and thyroid forms the anterior border of the retropharyngeal space. Bounded posteriorly by the alar fascia, the retropharyngeal space is limited laterally by the carotid sheaths and parapharyngeal spaces. The pretracheal, parapharyngeal, and retropharyngeal spaces communicate with each other. These spaces communicate with the subcutaneous spaces of the neck via the submandibular space and the mediastinal space. Because of these connections, infections or blood can track these spaces and a retropharyngeal hematoma can manifest itself as subcutaneous bruising. “Capp’s triad” describes the classical manifestation including compression of the trachea and esophagus, displacement of the trachea anteriorly, and subcutaneous bruising over the neck [9].

In the emergency of a spontaneous retropharyngeal hematoma it is crucial to decide which interventions are necessary. Symptoms may include dyspnea, dysphagia, or hoarseness. However, patients with early retropharyngeal hematoma can be seen with a sore throat without shortness of breath and may be misdiagnosed with a harmless viral pharyngitis. The protection of the airway has highest priority to maintain a sufficient respiration. In this context it is important to know if the size of the retropharyngeal hematoma is stable or progressive, because that information is important to assess the obstruction of the upper airway. Deaths can occur due to rapid development of respiratory distress from upper airway obstruction or great vessel compression caused by sublingual, retropharyngeal, and parapharyngeal hemorrhages [8]. Often the evacuation of the hematoma is not necessary to treat such patients successfully as the natural absorption of the hematoma is mostly sufficient. No need to say that continuous monitoring of vital parameters is obligatory. If respiration is insufficient even under a therapy with oxygen, then intubation or tracheostomy should be considered. It should also be considered that a transoral intubation could possibly lance the hematoma and cause severe bleedings. Obviously, parameters such as hemostasis and blood pressure should be optimized.

We hypothesize that an untreated OSA can cause spontaneous retropharyngeal hematoma. This could be due to the accompanying hypertension, the vibrations of the pharyngeal mucosa, or tissue alterations due to a chronic edema and inflammation of the subepithelial layers in upper airway tissues and the highly negative inspiratory pharyngeal pressures of down to − 100 cm H_2_O during obstructive apneas [10, 11]. Woodson *et al.* showed that histologic differences occur in snorers and in patients with OSA compared with non-snorers, visible as focal atrophy of muscle fibers, disrupted adjacent muscle bundles by infiltrating mucous glands, and extensive edema of the lamina propria with vascular congestion and dilatation [12]. Sometimes first clinical symptoms occur several hours after the precipitating event of such a hematoma. This may be the explanation of why our patient reported her first complaints several hours after getting up from bed.

Different studies showed that OSA can lead to several tissue alterations both in mucosa and subepithelial layers. Inflammation and edema of the subepithelial layers can lead to a loss of substance and damage to small vessels [10–12]. A blood vessel could tear at the junction of the mobile and the fixed parts of arteries or veins. This could lead to a spontaneous hemorrhage in the retropharyngeal space. It has already been shown that moderate to severe OSA can be one of the independent predictors of cerebral microbleeds which are considered a surrogate marker of overt stroke [13]. It is likely that OSA is also a predictor for bleedings in other compartments of the body.

If other causes for a spontaneous retropharyngeal hematoma are unlikely, an untreated or not sufficiently treated OSA should be considered. As far as we know, this is the first case report describing retropharyngeal hematoma caused by the adverse effects of OSA.

## Data Availability

The data used and/or analyzed during this case report are available from the corresponding author on reasonable request.

## Abbreviations

CPAP: Continuous positive airway pressure
ENT: Ear, nose, and throat
ICU: Intensive care unit
OSA: Obstructive sleep apnea
